# Bioinorganic Modulators of Ferroptosis: A Review of Recent Findings

**DOI:** 10.3390/ijms24043634

**Published:** 2023-02-11

**Authors:** Adrian Bartos, Joanna Sikora

**Affiliations:** Department of Bioinorganic Chemistry, Faculty of Pharmacy, Medical University of Lodz, Jana Muszynskiego 1, 90-151 Lodz, Poland

**Keywords:** ferroptosis, iron, inorganic, bioinorganic, inducer, inhibitor, modulator

## Abstract

Ferroptosis was first reported as a separate modality of regulated cell death in 2008 and distinguished under its current name in 2012 after it was first induced with erastin. In the following decade, multiple other chemical agents were researched for their pro- or anti-ferroptotic properties. Complex organic structures with numerous aromatic moieties make up the majority of this list. This review fills a more overlooked niche by gathering, outlining and setting out conclusions regarding less prominent cases of ferroptosis induced by bioinorganic compounds and reported on within the last few years. The article contains a short summary of the application of bioinorganic chemicals based on gallium, several chalcogens, transition metals and elements known as human toxicants used for the purpose of evoking ferroptotic cell death in vitro or in vivo. These are used in the form of free ions, salts, chelates, gaseous and solid oxides or nanoparticles. Knowledge of how exactly these modulators promote or inhibit ferroptosis could be beneficial in the context of future therapies aimed against cancer or neurodegenerative diseases, respectively.

## 1. Introduction

It was the year 2008 when Marcus Conrad and colleagues reported a new form of cell death associated with ROS-sensitive GPx4 protein (glutathione peroxidase 4) and interconnected with the metabolism of polyunsaturated fatty acids [1]. Stockwell and colleagues were the first to refer to it as ‘ferroptosis’ in their article published in 2012 [2]. Ten years later, ferroptosis can no longer be described as a poorly explored, obscure natural phenomenon, because the growing attention it draws from researchers is reflected in a multitude of published reports. In this short review, a number of newly reported ferroptotic inorganic inducers are listed and summarized in light of their mechanism of action and points of target. Inducers of both pro- and anti-ferroptotic effect are discussed simultaneously because modulation in either direction could be considered of therapeutic benefit. In the case of persistent cancer cases, ferroptosis would serve as a desirable tool to trigger targeted regulated death [3], much like apoptosis [4]. The issue is not exclusively pertinent to cancer treatment though. Inducers like artemisins may be used as inhibitors of osteoporosis due to their capability to induce ferroptosis in osteoclasts [5]. In cases like these, inducible ferroptosis becomes a tool to control the risk related to undesired cell survival. Alternatively, suppressed ferroptosis could prevent adverse diseases where intact cell survival is compromised due to progressing ferroptotic-type alterations, as is the case with diabetic nephropathy or non-alcoholic steatohepatitis [6]. In such cases, the accumulating intracellular changes and loss of cell viability are of greater concern than their possible uncontrollable proliferation. Whether pro- or anti-ferroptotic, bioinorganic compounds are more available from natural sources and easier to synthesize or purify than their organic counterparts. Some of these compounds are already mass-produced for various industrial purposes and hence are readily accessible. With the exception of physiological iron-based signaling, a diverse spectrum of these agents as potential modulators of ferroptosis are discussed below. The presented findings are preceded by a short outline of the ferroptosis mechanism that explains relevant highlights of the process and its critical points.

## 2. Concise Mechanism of Ferroptotic Progression

Ferroptosis, a non-apoptotic modality of regulated cell death [7] is linked to a number of inter-connected cellular events common to regulating oxygen metabolism and toxicity. The molecular processes precluding and contributing to the fatal outcome include the following components:

### 2.1. The Key Role of Iron

As the name suggests, the course of ferroptosis is highly dependent on intracellular labile ionic ferrous iron. The divalent metal directly fuels ferroptosis by reacting with cellular hydrogen peroxide in the course of the Fenton reaction. As a result, a much more unstable hydroxyl radical is produced, which causes oxidative stress in cells. Both iron [8] and hydrogen peroxide [9] are physiologically essential, but imbalance between the two becomes a threat to the redox equilibrium in cell.

Fe^2+^ + H_2_O_2_ → ^•^OH + OH^−^ + Fe^3+^

The release of iron from its storage macromolecule ferritin may be induced by the accumulation of ROS and further add to the oxidative stress [10]. The use of iron chelating agents typically reverts adverse changes, however it remains unclear if Fe overload is enough to initiate cell death mechanism [11]. Several other chemical elements can also mimic Fe and contribute to an even more efficient ROS generation through Fenton-like reactions [12]. Some hypothesize that other ferroptotic-inducing routes may exist independent of iron metabolism, which will be discussed below.

Fe^3+^ + H_2_O_2_ → Fe^2+^ + HOO

Ferric iron can also react with hydrogen peroxide to form hydroperoxyl radicals. Phospholipid hydroperoxyls are a substrate to GPx4 in mammalian cells [13], while their accumulation in cell is conductive to compromising its membrane integrity. Two fatty acids—arachidonoyl (AA) and adrenoyl (AdA), components of phosphatidylethanolamines, a class of phospholipids—are particularly susceptible to hydroxy-peroxidation [14,15].

### 2.2. Increased Formation of Reactive Oxygen Species (ROS)

ROS are unstable, short-lived, reactive, basal-level signaling molecules or by-products of cellular metabolism, the excess of which is highly toxic to the cell [16]. ROS include free radicals like superoxide anion (O_2_^•−^) or hydroxyl radical (^•^OH) and non-radical derivatives of oxygen—hydrogen peroxide (H_2_O_2_), hydroxide ion (OH^–^) or organic peroxides (ROOH) [17,18]. Their physiological homeostasis is susceptible to imbalance caused by numerous environmental stress conditions that can disturb natural ROS scavenging processes [19]. As many as 90% of ROS originate from leaking of the mitochondrial electron transport chain followed by much more reliable enzymatic sources [20]. Enzymatic and non-enzymatic defense systems exist. The former are metalloproteins that execute the multi-step process of converting adverse radicals to stable products (superoxide dismutases, catalase, glutathione peroxidases, peroxiredoxins, and thioredoxins). The latter interrupt free radical chain reactions (vitamins E, C, and A; glutathione, uric acid, plant polyphenol, carotenoids) [20,21,22]. When the level of free radicals overwhelms physiological antioxidant defenses, the cell undergoes oxidative stress as its intracellular susceptible biomolecules are exposed to damage [23,24].

Iron in not solely responsible for the emergence of reactive oxygen species. There are up to 12 mitochondrial enzymes engaged in nutrient oxidation that also give rise to endogeneous ROS [25]. The production of ROS takes place in various cellular locations and involves the activity of proteins such as: the family of nicotinamide adenine dinucleotide phosphate (NADPH) oxidases (NOX), specialized in the physiological formation of superoxide or hydrogen peroxide, which play a role in various types of host defense and the promotion of inflammatory response [26]; several oxidases based in peroxisomes, where up to 35% of total hydrogen peroxide generation occurs in mammalian tissues [27]; or the cytochrome P450 (CYP) family of enzymes implicated in the metabolism of xenobiotics [28]. Overall, as many as 40 enzymes are known to contribute to the emergence of ROS in cells, alongside non-enzymatic processes like the mitochondrial electron transport chain with its 11 identified distinct sites of electron leak [29].

### 2.3. Compromised Cellular Defense against ROS

One of the most critical points of ferroptosis involves the cystine/glutamate antiporter known as system Xc^−^. Its purpose is to import cystine and export glutamate simultaneously at a ratio of 1:1 in order to preserve glutamate homeostasis, satisfy the need for cysteine precursors and ensure the undisturbed synthesis of glutathione [30]. The system is composed of disulfide-linked heterodimers—family 7 member 11 and family 3 member 2 of the transmembrane transporter solute carrier (SLC) family, i.e., SLC7A11 and SLC3A2 respectively [31]. A dysregulated antiporter disables the synthesis of glutathione (GSH)—a potent antioxidant tripeptide composed of glycine, cysteine, and glutamic acid residues. In turn, the deficit of free GSH and inactivity of GSH-dependent apoenzymes marked by antioxidant properties, such as selenoperoxidase Glutathione Peroxidase 4 (GPx4), leads to oxidative stress caused by accumulating ROS [32,33]. The GSH/GPx4 defense axis is one of many used against ROS accumulation [34], but it is one of the most commonly mentioned in reports on ferroptosis [35].

### 2.4. Target of ROS: PUFA Metabolism

Lipid peroxides are formed from polyunsaturated fatty acids (PUFAs, components of phospholipids found in mammalian cell membranes) oxidized by ROS. Phosphatidylethanolamines, which make up 25% of phospholipids in cells [36], are the first to undergo oxidation [31]. Idle GPx4 fails to reduce accumulating phospholipid hydroperoxides to non-toxic lipid alcohols, which further favors disruption of cell membranes and the resultant ferroptotic fate [37,38]. GPx4-independent mechanisms of lipid peroxide reduction exist, including NADH-FSP1 (ferroptosis suppressor protein 1)-CoQ1, and GCH1 (GTP cyclohydrolase-1)-BH4, the disruption of which also promotes ferroptosis [39]. When GPx4 is scarce, a dihydroorotate dehydrogenase (DHODH) mediated mechanism serves as a secondary regulator, as it is capable of aggravating or somewhat overriding the ferroptotic response, depending on its enzymatic substrate/product ratio [40]. Enzymes involved in PUFA-containing phospholipids biosynthesis, such as ACSL4, can be inactivated to rescue cells from lethal alterations [41].

As a result of these numerous interconnected cellular dysfunctions, ferroptosis manifests in such morphological features as smaller mitochondria, diminished or vanished mitochondria crista and condensed mitochondrial membrane densities [42]. Unlike apoptosis, no dedicated signalling pathway directly leads to ferroptosis. Instead, the fatal outcome is a consequence of certain tightly regulated physiological processes being specifically distorted. Ferroptosis is therefore considered a regulated type of cell death, rather than a programmed one, but it is still far from spontaneous and haphazard, as is the case with necrosis. Notably, the programming involves the recruitment of iron prior to ferroptosis through upregulation or activation of proteins involved in iron metabolism, i.e.: divalent metal transporter 1 (DMT1), an iron transporter that carries dietary iron into enterocytes; ferroportin, which transports iron from enterocytes to the blood; transferrin receptor 1 (TfR1), which is required to transport iron to peripheral tissues; or NCOA4, whichmediates recruitment of ferritin for autophagy [43].

Because the multiple signalling nodes associated with ferroptosis serve a multitude of functions, and are scattered across various cell locations and physiological processes, it seems incorrect to refer to this process as a *pathway* per se. Its induction or inhibition can be achieved by targeting the pool of intracellular iron, ROS, antioxidants, the Xc^−^ system as well as systems responsible for membrane repair or composition. Hence, when looking to define what ferroptosis is, it should be thought of as a vulnerability to lipid peroxidation by reactive iron species, which can be potentiated or patched at several distinct stages of cell death progression.

**Figure 1:**
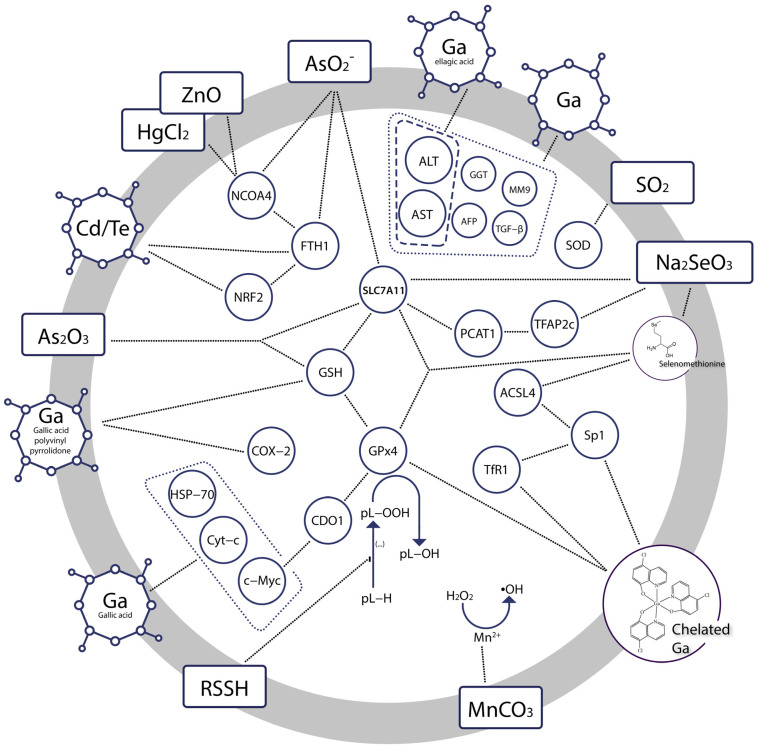
Molecular effectors associated with ferroptotic induction by bioinorganic compounds. Abbreviations: alanine transferase (ALT), aspartate transferase (AST), gamma-glutamyltransferase (GGT), alpha-fetoprotein (AFP), metalloproteinase-9 (MM-9), transforming growth factor β (TGF-β), superoxide dismutase (SOD), transcription factor AP-2 gamma (TFAP2c), prostate cancer associated transcript 1 (PCAT1), acyl-CoA synthetase long chain family member 4 (ACSL4), specificity factor 1 (Sp1), transferrin receptor 1 (TfR1), solute carrier family 7 member 11 (SLC7A11), glutathione (GSH), glutathione peroxidase 4 (GPx4), phospholipid (pL), heat shock protein 70 (HSP-70), cytochrome c (Cyt-c), cellular myelocytomatosis oncogene (c-Myc), cysteine dioxygenase type 1 (CDO1), prostaglandin-endoperoxide synthase 2 or cyclooxygenase 2 (COX-2), nuclear factor erythroid 2–related factor 2 (NRF2), ferritin heavy chain 1 (FTH1), nuclear receptor coactivator 4 (NCOA4).

Some of the known ferroptotic modulators with such functions include iron chelators [44], talaroconvolutin A [45], RAS-selective lethal 3 (RSL3) [46], erastin [47] and ACSL4 inhibitors [48], respectively. Inducible ferroptosis could potentially become a subject of pharmaceutical strategy [49], because it could help circumvent the resistance reported in cases of cancer that are refractory to therapies based on apoptosis [50]. To this end, compounds are tested for potency to drive cells towards ferroptosis or, alternatively, avert them from the path of this regulated cell death. The most popular of today’s known chemical inducers that affect the development of ferroptosis include complex organic molecules, whether natural or synthetic, with multiple aromatic moieties, like erastin, FIN56 or RSL3 to name a few [51]. A lot less attention is drawn to non-carbon based compounds, which are oftentimes much simpler and more readily available for research purposes. Figure 1 outlines some of the identified molecular interactions caused by bioinorganic modulators of ferroptosis. The impact and significance of the chemical compounds selected for this review is then described in thorough detail.

## 3. Gallium Salts, Complexes and Nanoparticles

Iron chelation is a known strategy for the prevention of iron-dependent ferroptosis. Ferroptotic changes were shown to withdraw upon the application of chelating agents such as deferiprone, deferoxamine [52], 2,2′-pyridine [53] or ciclopirox olamine [54]. Otherwise, the availability of the labile metal can be modulated with what can be regarded as an antagonist of cellular iron-binding components. Gallium ions exhibit a notable chemical resemblance to iron and are capable of superseding it within its molecular complexes [55]. Under physiological conditions, they are unable to change into +2 oxidation state. Iron-dependent molecules bound to redox-inactive gallium therefore lose their potency and interrupt the respective signalling pathways they take part in [56]. The oxygen uptake of heme molecules, however, remains intact [57].

The disruption of iron metabolism using gallium is thought to be a cancer treatment tool based on the ferroptosis mechanism. This is an especially promising direction given the higher demand for iron in rapidly proliferating tumor cells, which adds to the specificity of such an approach [55,58]. Gallium nitrate was a first generation therapeutic approach against cancer. Its citrate-buffered solution was approved by the United States Food and Drug Administration (FDA) as a form of treatment for malignant tumor-related hypercalcemia [59]. Clinical trials also point to antineoplastic activity against bladder cancer and non-Hodgkin’s lymphoma [60]. Treatment with gallium nitrate does not suppress hematopoiesis [60], but its drawback is that upon administration the compound is prone to hydrolysis, forming insoluble oxides, which results in reduced transmembrane delivery and efficacy of the product [61]. Following the success of this chemotherapeutic, a number of other gallium-based potential drugs were developed [62], including gallium maltolate (tris(3-hydroxy-2-methyl-4H-pyran-4 onato)gallium(III)) and KP46 (tris(8-quinolinolato)gallium(III)). Many of these precede the notion of ferroptosis and thus were not researched specifically in this context. Instead, compounds were ascribed general anti-tumor activity and a reported interplay with select molecular elements of what is today known as ferroptosis [63,64,65]. The link between gallium-disturbed iron metabolism and ferroptotic regulated cell death has only been the subject of research in the last few years.

The most notable improvement to gallium nitrate was to apply the metal element as 1–100 nm-sized nanoparticles marked by chemical and physical differences advantageous over its bulk form. An orally administered therapeutic combined with low-level gamma radiation treatment exhibited antitumor properties in hepatocellular carcinoma induced in rats with diethylnitrosamine. The antineoplastic action of gallium was considered to be a consequence of its capacity to disrupt tumor cell iron homeostasis [66]. In the following years, gallium nanoparticles (GaNPs) would often be combined with various other compounds to which they served as a synergist/additive as well as carrier matrix. The molecules affected by these constructs were increasingly associated with ferroptotic cell death.

Gallic acid (GA) is generally linked to apoptosis, but cells induced with GA do not manifest exclusively apoptotic symptoms. Reported nuclear condensation, plasma membrane rupture and lipid peroxidation were indicative of multiple simultaneous cell death types induced, such as apoptosis, necroptosis and ferroptosis in response to incubation with GA. Despite targeting all three processes with specific inhibitors, GA-induced cell death persisted and could only be suppressed with deferoxamine, suggesting an iron-dependent mechanism [67]. Hydrophillic gallic acid is, however, flawed by poor permeability through membranes of cancer cells, which limits its therapeutic use. To bypass this problem, gallic acid-coated gallium nanoparticles were applied against the human hepatocellular cancer (HepG-2) cell line [68]. With the incorporation of gallium, the solution could also potentially narrow down the number of induced modalities of cell death. GA-GaNPs at the size of 40 to100 nm exhibited greater cytotoxicity than cisplatin with over four-fold lower IC_50_ and modulated expression of c-Myc (cellular myelocytomatosis oncogene), HSP-70 (heat shock protein-70) and Cyt-c (cytochrome c) at a comparable level to that of CDDP (cisplatin). All these proteins are known to facilitate the occurrence of ferroptosis. C-Myc regulates cysteine dioxygenase type 1 (CDO1) [69], the inhibition of which restores the activity of GPx4 [70]. Anti-apoptotic protein HSP-70 is involved in rendering resistance to multiple types of regulated cell death [71,72] and was shown to sustain damage from increased levels of ROS and lipid peroxides [73]. Enzymatic oxidation of cytochrome c, on the other hand, promotes ferroptosis caused i.a. by the accompanying formation of cytotoxic superoxide and hydroxyl radicals [74]. Apart from its protein regulatory effect, gallic-acid-coated gallium nanoparticles also displayed homeostatic properties with respect to intracellular iron content and antioxidant enzyme activities, including glutathione [68], which further proves the ferroptosis-modulating character of gallium.

Adding to the versatility of gallium-based therapy, nanoparticles composed of gallium nitrate were combined with naturally occurring polyphenol ellagic acid, known for its antioxidant activity and radical scavenging capacity. The cytotoxicity of this preparation on the human breast cancer (MCF-7) cell line cultured in vitro was expressed with IC_50_ at 2.86 ± 0.3 μg/mL. It was also confirmed as hepatoprotective and renoprotective in the course of in vivo testing against 7, 12 dimethylbenz[a] anthracene (DMBA)-induced mammary gland carcinogenesis in rats. Yet again, the authors did not specifically investigate the possible ferroptotic character of the reported cellular response, focusing on how efficient the antitumor activity was, but a few relevant observations can be made [75]. The nanoparticles decreased the level of alanine transaminase and aspartate aminotransferase. The same effect was observed in a different study where bare GaNPs were employed [66], suggesting this was a direct effect of the gallium rather than ellagic acid. Interestingly, the demise of the two enzymes in response to gallium was also reported in the course of acetaminophen-induced liver injury, postulated to rely on ferroptosis and being somewhat susceptible to fer-1 inhibition [76]. Other than that, the administration of gallium–ellagic acid nanoparticles caused an increase of GSH and superoxide dismutase, the latter being one of the major elements of cellular protection against ROS-mediated oxidative damage [77]. Less malondialdehyde, on the other hand, was indicative of an inhibited lipid peroxidation process in cancer cells [78]. The gallium atoms likely contributed to normalizing the serum level of iron, as reflected by lower total iron-binding capacity (TIBC) relative to the DMBA iron-deficient group, where much of iron supply was consumed by malignant cells [75]. How much of the reported antitumor effect was related to the ferroptosis-inducing properties of the gallium-based agent remains up for debate. However, the idea of a relationship between exposure to gallium and ferroptotic cell death is becoming increasingly substantiated.

The mutual interplay between the two was more evident in a study on kidney injury pathogenesis. Given that certain clues of the medical condition are somewhat associated with iron homeostasis, gallic acid-gallium polyvinyl pyrrolidone nanoparticles were used to target iron metabolism and lipid peroxidation in HK-2 (human kidney 2) cells. Treatment with nanoparticles aimed against ferroptosis partially recovered both the morphology and the viability of the cells and brought about a comparable outcome to ferrostatin-1, a ferroptosis inhibitor. The effect is said to be otherwise unachievable with the use of free gallic acid due to the low concentrations applicable without nanoparticles. Other improved cytoprotective parameters pointing to the regression of ferroptosis included: downregulated expression of prostaglandin-endoperoxide synthase 2, a ferroptotic marker; restored mitochondrial membrane potential and oxygen consumption rate; suppressed mitochondrial ROS accumulation and cellular lipid peroxidation; rebalanced intracellular GSH and free iron levels; and retrieved GPX4 protein activity. Overall, the nanoparticles were successful at mitigating renal tubular injury in vivo, which is thought to rely on the ferroptosis mechanism and leads to acute kidney injury. The suppression of ferroptosis may thus serve as a strategy to prevent and treat renal damage [79].

Various other, more complex gallium compounds can be synthesized to serve as potentially enhanced ferroptotic modulators. Gallium coupled with an organic ligand–cloxyquin (5-chloro-8-quinolinol, HClQ) was shown to be an even more specific inducer of ferroptosis in rhabdomyosarcoma cells. Used separately, both molecules are established as effective therapeutics. Some of the properties attributed to HClQ include anti-melanoma and p53-modulating effects. Cells incubated with the free compound display both early necrotic and early apoptotic morphologies lacking any molecular hallmarks of ferroptosis. When incubated with Ga(III)-HClQ, non-apoptotic cell death was reported in cancer cells, which was precluded in the presence of ferrostatin, an inhibitor of ferroptosis, as well as IM-54, an inhibitor of oxidative stress-induced necrotic cell death. Cells were marked with a declined level of GPx4 as well as elevated levels of ROS and lipid peroxides. Ferroptosis was in this case considered the prevalent type of reported cell death, followed by autophagy; the induction of the latter can be associated with the effect caused by free HClQ [80].

The use of gallium for therapeutic applications holds significant promise due to its nearly unique fluidic and metallic properties. Liquid metal particles are apt for greater surface functionalization, mechanical manipulation and integration with a range of organic polymers and inorganic components [81]. The aforementioned findings in relation to gallium-based compounds and their effects are recapitulated in Table 1 below.

## 4. Chalcogens: Sulphur, Selenium, Tellurium

Thiols (RSH) [82] and—to a much greater extent—hydropersulfides (RSSH) [83] have lipophilic radical-trapping antioxidant properties, which helps prevent lipid peroxidation and ultimately averts ferroptotic damage to the cell. While these inorganic compounds are mostly engaged within intracellular signaling pathways in the ongoing cell death process [84], some of reports emerged in recent years emphasizing the significance of a double sulphur-based group that produces an anti-ferroptotic effect. To circumvent the instability of the short-lived, self-reacting hydropersulfides, an organic cumyl-SSH molecule (an *N*-alkylamine perthiocarbamate derivative) was synthesized as a precursor capable of releasing RSSH in situ. The gradual introduction of hydropersulfide from the internalized decomposing agent reverted RSL3-induced ferroptosis in Pfa1 mouse embryonic fibroblasts with EC_50_ = 6.2 μM over the 6-h incubation period [83]. Despite the fact that the reactivity of RSSH towards phospholipid-derived peroxyl radicals was on par with many known ferroptosis inhibitors, such as Fer-1 and Lip-1, its antiferroptotic potency reported in a biological system fell behind. Suplhur compounds, however, demonstrated potential for suppressing iron-dependent cell death.

The significance of hydropersulfides was further confirmed by Dick et al. [85] who reported the ability of hydropersulfides to scavenge free radicals over the three-step autocatalytic cycle in which persulfides are regenerated. The chemical reaction is conducive to the suppression of lipid peroxidation, and ultimately inhibits ferroptosis. In fact, the emergence of the intracellular sulfane sulfur (S^0^) species, including hydropersulfides, hydropolysulfides and polysulfides, is part of an adaptive response to pro-ferroptotic induction. Their cytoprotective effect is so notable, that S^0^ biosynthetic pathways can be perceived as a parallel defense axis. This is also a new look at cysteine residue, which is no longer solely a GSH component, but now becomes a source of sulfur for S^0^ species, highly potent antioxidants that prevent ferroptosis independently of the most canonical GPx4 pathway. Endogenous compounds involved in S^0^ metabolism, as well as exogenous supplementation with membrane-permeable S^0^ species, facilitate manipulation of the ferroptotic cell fate. By tuning the activity of two enzymes of opposing properties—cystathionine γ-lyase, which supports the biosynthesis of persulfides, and persulfide dioxygenase, which degrades them—the authors were able to modulate the removal of endogenously produced free radicals and switch between different states of vulnerability to lipid peroxidation.

Sulfur dioxide, on the other hand, causes substantial oxidative stress in cells which can serve to induce ferroptosis. An experimental prodrug was created based on this idea with an intended application against gastric cancer. The structure of amphiphilic polimer included 2,4-dinitrobenzenesulfonamide, the decomposition of which results in the release SO_2_. Upon its internalization into cells, the compound caused a dual induction of ferroptosis based firstly on the reported depletion of GSH, leading to downregulation of GSH-dependent GPX4, and secondly on the promotion of ROS-generation as a fuel for lipid peroxidation. The inhibition of tumor growth caused by SO_2_ increased the cancer cell death rate 10-fold and could target cancer cells in a specific manner. The release of gas from the organic matrix required prior stimulation from intracellular thiols. This was enabled by the abnormally increased GSH pool, a feature of cells undergoing cancer progression. Cell mortality rate was then restored with the use of ferrostatin-1 and deferoxamine [86].

Selenium has been long known as an apoptosis inducer through superoxide generation in cells [87], which ultimately raised the question of the possible application of this element in cancer treatment and its hypothesized participation in ferroptosis. The implication of selenium in ferroptosis seems evident given: (i) its chemical similarity to sulphur [88], as was the case between iron and gallium; (ii) the selenoenzymatic nature of glutathione peroxidase 4 protein (GPX4), the main regulator of membrane-associated lipid peroxidation [86]; (iii) the uptake and accumulation of selenium being correlated to the x_c_^−^ antiporter system known to be one of the gateways to developing ferroptosis [89]; and (iv) a long list of anti-cancer effects ascribed to numerous selenium-based compounds, either with or without acknowledgement of ferroptosis as a distinct mechanism of cell death being induced [90]. Over the years, inorganic and organic selenium-based compounds of varying degrees of toxicity were tested for their application as therapeutic selenium donors.

Indeed, sodium selenite was shown to induce regulated cell death by disturbing a number of the most fundamental elements of ferroptosis, including SLC7A11, GSH and GPx4, which inevitably led to ROS generation and lipid peroxidation in human cancer cells. Once again, Fer-1 and DFO suppressed the process, as did the expression of antioxidant enzymes [91]. However, systemic exposure to selenium, even at seemingly ordinary intake levels, remains a controversial subject with contradictory results in clinical trials regarding the risk of developing cancer, neurodegenerative diseases or type 2 diabetes [92]. Inorganic selenium compounds, thought to be a possible anti-cancer remedy, may actually yield an overall opposite effect [93] despite their environmental ubiquity and functional importance [94].

Once taken up and assimilated, the most common inorganic forms of selenium, i.e. sodium selenite and selenite, are typically subject to intracellular chemical conversion. In yeasts, the compounds are converted into organic selenomethionine, the properties of which were shown to retain the ROS-inducing effect coupled to the element. Exposure to the compound was shown to cause morphological defects of embryonic eye development in zebrafish. Alongside apoptosis, it was ferroptosis that was found to be one of the underlying mechanisms of the Se-induced retinal cell death, as evidenced by condensed mitochondria, iron overload in cytoplasm, a decrease in GSH levels, and an increase in malondialdehyde levels. However, many ferroptosis-related genes, i.e. acsl4, gpx4 and slc7a11, were dysregulated, presumably disrupting control over the cell death process. Contrary to numerous in vitro studies where ferrostatin-1 is routinely reported to rescue cultured ferroptotic cells, herein the authors did not register macroscopic alleviation of ocular defects in the selenium-stressed embryos, despite Fer-1 supplementation and its clear impact on ferroptosis-related gene expression. Instead, some of the ocular defects caused by selenium stress were to a large extent withdrawn upon treatment with cisplatin, a ferroptosis/apoptosis activator. CDDP also normalized the expression of acsl4 and gpx4, ensuring more coordinated or selective ferroptosis. The results point to the regulation of ferroptosis as one of the possible response mechanisms to excessive selenium [95].

In mammals, on the other hand, inorganic forms of selenium such as selenite and selenate are converted intracellularly to selenide, which can be further converted to selenophosphate and used to generate selenocysteine, which is used for protein synthesis of selenoproteins, such as GPx4 [96].

In opposition to the above-referenced pro-ferroptotic properties of sodium selenite and the product of its bioconversion, a different report suggests anti-ferroptotic activity by these compounds. Both inorganic forms of selenium–selenite and selenate proved efficient at rendering resistance in the N27 immortalized neuronal cell line against erastin-induced ferroptosis with IC_50_ values of 38 and 925 nM, respectively, and against RSL3-induced ferroptosis with IC_50_ values of 15 and 489 nM, respectively [97]. While in the first report [91], sodium selenite at concentrations of 2.5–12.5 µM was shown to decrease the GPx4 level up to 10-fold in MCF-7 cells, more than 2-fold in PC3 cells and nearly 3-fold in U87MG cells upon 1–2 h treatment, the results found in report [97] indicated an approximately 3-fold increase in GPX activity upon 24-h treatment of N27 cells with 1 μM selenite salt as well as a 3-fold increase in GPx4 protein level in cells. What emerges out of these results is that sodium selenite can indeed be used as a selenium donor to boost selenoprotein levels but care has to be taken, as high levels of the element induce superoxide and may lead to lipid peroxidation. The toxicity of a specific ferroptotic Se-based modulator largely determines the direction of the cellular fate to be attained. Benign forms of pharmacological selenium, such as brain-penetrant selenocysteine peptides, have great potential as neuroprotective agents in the treatment of intracerebral hemorrhage or Parkinson’s disease [98].

In a related study, an intracerebroventricular administration of 3 µL sodium selenite at 2.5 µM was shown to also evoke an inhibitory effect against ferroptosis in murine spinal cord tissues. The progression of spinal cord injury was directly linked to ferroptotic cell death, and the neuroprotective effect of sodium selenite as a ferroptosis suppressor was substantiated by alleviated iron overload, increased GSH and GPX4 expression, decreased levels of lipid peroxidation and the recovery of typical cellular manifestations of ferroptosis observed microscopically [99]. Similar effects were observed in the course of another type of neuronal injury, intracerebral hemorrhage. This type of stroke results in neuronal death by ferroptosis beginning shortly after. Regulated cell death can be inhibited in a dose-dependent manner with the administration of sodium selenite. Selenium at 1 μM introduced 2 h post the onset of chemically induced ferroptosis preserves 80% viability of primary cortical neuron culture. A twelve-hour delay resulted in 20% neuroprotection. GPx4 was identified as one of the multiple mediators of upstream selenium exposure. The transcriptional response to the element involves upregulation of nuclear and mitochondrial GPx4, while suppression of the protein renders Se-induction dramatically less efficient at preventing cell death. Other selenium-sensitive proteins include TFAP2c and Sp1, transcription factors belonging to what was dubbed *selenome*. These are however only two of 238 known Se-inducible genes and most likely not the only ones to mediate ferroptosis [98]. TFAP2c was shown to act as a ferroptosis inhibitor by activating prostate cancer-associated transcript 1 (PCAT1), the latter being an activator of SLC7A11 expression [100]. The role of Sp1, on the other hand, is a little more ambiguous in the context of ferroptosis. It was shown to upregulate the transcriptional activation of Prdx6, the latter being an inhibitor of high glucose-induced ferroptosis in the mouse glomerular podocytes [101]. At the same time, Sp1 is known to promote the expression of ACSL4 (Acyl-CoA synthetase long-chain family member 4), which in turn targets certain PUFAs to produce their Co-A derivatives. As such, they are subject to consecutive chemical modifications until lipid hydroperoxide is produced [102,103]. Sp1 is also capable of upregulating TFRC transcription, which encodes transferrin receptor 1 (TFR1), a protein implicated in the intracellular iron levels homeostasis. As a result of the overexpression of TFR1, excessive uptake of intracellular iron occurs, leading to ferroptosis [104]. The reported Se-induced activation of anti-ferroptotic GPx4 by means of possibly pro-ferroptotic Sp1 requires further insight.

Another chalcogen, tellurium, was used in combination with cadmium as a reactive crystalline core in semiconductor nanocrystals, also known as quantum dots. RAW264.7 macrophages exposed to 1 μM CdTe crystals exhibited ferroptosis. Upon induction, it was found that ferroptotic signaling involved steps such as intracellular depletion of nuclear factor erythroid 2-related factor 2 (NRF2), phosphorylation of extracellular regulated protein kinases1/2 (ERK1/2) and degradation of ferritin heavy chain 1 (FTH1), the major iron storage protein [105]. The reported decrease in NRF2 in cells translates into lower expression of SLC7A11, which impairs the Xc- cystine uptake system. However, the causality of ferroptosis in this case in not linear, with multiple simultaneous pathways affected that lead towards the same outcome. The NRF2-dependent activation of transcription impacts over 300 genes, including: heme oxygenase-1, the product of which contributes to the release of free iron and is hypothesized to participate in regulating intracellular iron metabolism [106]; quinone oxidoreductase 1, the light and heavy chains (FTL/FTH1) of the iron storage protein ferritin [107]; metallothionein 1G, which yields ferroptosis resistance [108]; and genes like NQO1, AKR1C1 and GST that are marked by their *antioxidant response element* binding sequence and participate in maintaining redox homeostasis [109], to name a few. The aforementioned findings relating to chalcogen-based compounds and their effects are recapitulated in Table 2 below.

## 5. Transition Metals

Manganese is more efficient than iron at catalyzing the conversion of hydrogen peroxide into hydroxyl radical. The Fenton-like reaction, however, is not nearly as broadly studied in the context of ferroptosis [110]. An intracellular influx of manganese ions is conducive to ferroptosis through excessive ROS formation and accumulation. Several delivery systems have been proposed to introduce this element at high volumes and overwhelm cellular antioxidant systems in the tumor environment. Manganese carbonate fabricated in the form of nanocubes would degrade in acidic pH and release Mn^2+^, which can then convert endogenous H_2_O_2_ into a much more toxic hydroxyl radical (•OH). While the exposure of cancer cells to reactive oxygen species is not a novel therapeutic approach, chemodynamic therapy tools like this one could successfully complement traditional photodynamic therapy [111]. Similarly, manganese dioxide was introduced into cancer cells on mesoporous silica nanoparticles. Upon its internalization, MnO_2_ works in a dual mode. Firstly, is it a source of internally released Mn^2+^ ions, causing oxidative stress through a Fenton-like reaction in conditions buffered by the physiological occurrence of bicarbonate/carbon dioxide. Secondly, the inorganic compound also stimulates sulphurization of glutathione, thus disrupting its ROS scavenging effect and disabling its related antioxidant defense mechanism [112]. In another study, researchers used an upgraded matrix prepared by hydrolization of L-arginine-capped SiO_2_ and binding it to manganese ions. The colloid mainly consisted of manganese silica and indicated residual Mn ions at various oxidation states, including MnO, Mn_2_O_3_, and MnO_2_. The arginine-rich surface layer enabled a tumor homing feature based on the commonly reported deficiency of argininosuccinate synthetase (ASS) in cancer cells. A novel physico–chemical structure, with the compound being dispersed in CO_2_ nanobubbles, allowed more rapid Mn^2+^ release and higher GSH depletion rates compared to solid-state nanoparticles. The resultant ferroptotic cell death occurred via indirect GPX4 inactivation followed by elevation of intracellular oxidative stress and ultimately lipid peroxidation [113]. All referenced experiments took advantage of tumor acidosis resultant from local deficiencies of oxygen within a substantial tumor mass. Hypoxic cells resort to glycolysis as their source of energy and metabolize available substrates to CO_2_ or H^+^ ions, creating a microenvironment of acidic pH [114]. The three attempts at inducing ferroptosis follow the approach of stimulating a Fenton reaction between hydrogen peroxide, a physiological product of mitochondrial oxidative respiration, and manganese ions purposefully introduced into the cell. To improve the efficiency of the potential drug, the pool of H_2_O_2_ available for the Fenton reaction can be extended beyond its endogenous level. In a case like this, iron oxide nanoparticles were layered with manganese carbonate and additionally conjugated with cisplatin prodrug. The latter is a Pt(IV)-based complex which, upon its entry to the tumor microenvironment, undergoes conversion to an anticancer drug containing Pt(II). It also upregulates intracellular H_2_O_2_, therefore stimulating the formation of hydroxyl radicals and simultaneously inducing ferroptosis in addition to apoptosis. Compared to cisplatin, the pH-responsive nanoplatform exhibited an equivalent antitumor effect while harnessing much less platinum, therefore reducing systemic toxicity of the preparation [115].

Manganese can be an alternative to iron in generating ROS, but so can be other metallic substrates like copper and cobalt, in which case the phenomenon is referred to as a Fenton-like reaction [116]. In fact, the alternatives may in certain ways be superior. Manganese carbonate was shown to exhibit greater sensitivity to the acidic environment than iron and is more pH-dependent, hence the selective release of Mn^2+^ [115], while copper ions were demonstrated to be much less constricted by pH conditions than iron when it comes to catalyzing Fenton-like reactions at high rates [117]. In the same way that pro-ferroptotic and apoptotic agents were combined in a referenced example, Fenton-reactive polymetallic drugs can be fabricated with the benefit of their synergy. Copper/manganese silicate nanospheres were based on the idea that one of the elements could additionally serve as a photosentitizer for hypoxic tumor cells, somewhat resistant to ROS generation by means of photodynamic therapy. The incorporation of copper relieved their oxygen deficiency through the Cu-catalyzed decomposition of endogenous H_2_O_2_ to O_2_. Upon external irradiation, these cells exhibited the conversion of newly formed molecular oxygen to singlet oxygen, one of the reactive oxygen species required for ferroptosis. Simultaneously, both copper and manganese acted as chemodynamic agents, degrading hydrogen peroxide to hydroxyl radical. Such a drug design allowed the spectrum of clinical tools used for anti-cancer therapy to widen [118].

Having found how a group of transition metals—Fenton-like inducers—is linked to ferroptosis, the focus of researchers is now undergoing a shift towards devising readily internalized, non-toxic carriers marked by features such as good drug-loading potential, sensitivity to subtle changes in microenvironment conditions, controllable biodegradability into cationic forms and drug release.

The role of transition metals in modulating ferroptotic death does not have to be limited to mimicking iron in Fenton-like reactions. Diacetyl-bis(4-methyl-3-thiosemicarbazonato)copper^II^ or Cu^2+^ (atsm) was shown to counteract ferroptosis and thereby bring improvement in the course of neurodegenerative diseases, such as lateral sclerosis and Parkinson’s disease, the progression of which is associated with ferroptotic cell death. The radical trapping activity of the compound was so pronounced that its resultant antiferroptotic potency was reported to be comparable with liproxstatin-1, which was used as positive control. It should be noted that CuSO_4_ left cells unaffected. The reported effect was therefore not resultant from simply increasing intracellular Cu concentration [119]. As for free copper ions, on the other hand, they are stimulatory to ferroptosis and this is neither a Fenton-dependent nor an iron metabolism-dependent interaction. Cu^2+^ augments lipid peroxidation and ferroptotic cell death through binding GPx4 protein cysteines C107 and C148. This in turn increases GPX4 ubiquitination and the formation of GPX4 aggregates, causing Tax1 binding protein 1 (TAX1BP1)-dependent autophagic degradation of this master regulator. Acute pancreatitis, associated with ferroptotic damage of cells, is likely to somewhat rely on copper ions and could be attenuated by copper chelator tetrathiomolybdate [120].

Zinc oxide, manufactured in the form of nanoparticles with diameters less than 100 nanometers, is used for numerous industrial applications including dental care [121], cosmetics [122] and nutrition [123]. Its popularity is far past the laboratory-scale stage with approximately 550 tonnes of the compound being produced worldwide every year [123]. Zinc oxide nanoparticles were confirmed as inducers of ferroptosis in endothelial cells, but the process was shown to be routed through the autophagy degradation system [124]. Autophagy is a conserved cell survival mechanism of degradation and recycling of cellular components (referred to as *autophagic cargo*), whether it is proteins or entire organelles, the breakdown of which helps maintain metabolic homeostasis in stress conditions [125]. Ferritinophagy is a specific type of autophagy that involves the degradation of ferritin, which unavoidably causes the release of large quantities of iron into cytosol [126].

Ferritinophagy-promoted ferroptosis is a readily exploited vulnerability that can be targeted with the use of compounds that act as inhibitors of iron translocation from lumen to cytosol. This causes iron-depletion stress, leading to lysosomal degradation of ferritin and lysosomal production of ROS [127]. Ferroptotic cell death by zinc oxide induction is a result of iron overload caused by the autophagic degradation of FTH1, a subunit of ferritin. The process is mediated by NCOA4 (nuclear receptor coactivator 4), a *cargo receptor* responsible for recognizing and tethering ferritin in the vesicles (autophagosomes) for their subsequent delivery into the degradation system [128]. Zn-induced effects were alleviated by impediments set up at every the stage of the pathway: ferroptotic death (general ferroptosis inhibitor—Ferrostatin-1, iron chelator—deferiprone), autophagy (inhibitors of fusion between autophagosomes and lysosomes—chloroquine and bafilomycin A_1_, autophagosome formation inhibitor 3-MA) and ferritinophagy (NCOA4 knockdown) [124].

What is noteworthy is that recent reports indicate that iron endocytosis may be a much more complex phenomenon than initially thought. Apart from engaging known iron metabolism regulators, including NCOA4, transferrin receptor 1 (TFR1), divalent metal transporter 1 (DMT1) and ferroportin (known as FPN or SLC40A1) [129], the process runs along a co-existing parallel pathway dependent on iron-bound Hyal, a principal ligand of CD44 transmembrane glycoprotein linked to epigenetic plasticity. In line with this finding, the role of iron and its potential mimetics extends far beyond ferroptosis induced in cancer cells and the element becomes a valid regulator of epigenetic plasticity [130]. Future therapies can potentially not only affect the cellular fate of the cell by dysregulating iron metabolism, as was already proved feasible by inhibiting DMT1 in cancer stem cells [131], but could also evoke a shift in a variety of processes accompanying cell transformation, including its development, inflammation, immune responses, wound healing and cancer progression [130].

Given the implication of zinc in the modulation of ferroptosis, a question arose whether zinc affects cell fate by its inherent mechanistic routes, which have to date been overlooked, or if it merely disturbs the iron metabolism of well-established significance. Arguments additionally reinforcing the role of zinc came from data indicating that inhibition of ZIP7 and ZIP14, the regulators of interorganellar zinc transport, prevented cells from undergoing ferroptotic death. Cases of ferroptosis reportedly lacking iron accumulation could in this context point to a possible alternative onset of Zn-dependent ferroptosis without iron being central to the process. As a Fenton-inactive metal, zinc would not rely on the already agreed on ROS generation start-up mechanism [132]. This notion can, however, be countered with the fact that disturbed intracellular zinc translocation entailed the activation of several already-identified genes that have a protective effect against ferroptosis [132,133,134]. Zn-induced cancer cells mirrored the mechanistic hallmarks of Fe-induced cells, including elevated ROS (despite zinc being a redox-inactive metal), lipid oxidation, depleted GSH and downregulated GPX4. While these effects could be reversed with zinc chelation, the same was observed upon treatment of cells with iron chelator desferrioxamine. ZnO nanoparticles were ultimately shown to affect the intracellular release and uptake of extracellular ferrous iron by downregulating the mRNA levels of ferritin subunits and upregulating the transcription of iron importers TFRC and DMT1. This leads us to believe that the cellular response to zinc remains iron-mediated [132,135]. In principle, ferroptosis occurs provided that there is lipid peroxidation and prevention of cell death is possible through the use of iron chelators and ferroptosis inhibitors. Remarks on ferroptosis as a regulated cell death centrally driven by anything other than iron metabolism are a contradiction in terms, unless an entirely different cell death modality is proposed instead.

The aforementioned findings relating to compounds formed by transition metals and their effects are recapitulated in Table 3 below.

Thus far, we have discussed multiple examples of bioinorganic compounds that impact iron metabolism and thus modulate ferroptotic death in somatic cells, which helps reduce the clinical risk of primary tumors. Incidentally, iron overload plays an even greater role in compromising the self-renewal of drug-tolerant stem cells, while iron deficiency inhibits their pluripotency [136]. The augmented sensitivity of the stem cell cycle to iron homeostasis may be of therapeutic significance in the context of relapsing cancers, while ferroptosis could be the key to the successful eradication of persister cancer stem cells. This is the case with ovarian cancer tumor-initiating cells, intrinsically marked by decreased expression of ferroportin (FPN), an iron efflux pump, and increased expression of transferrin receptor (TfR1), an iron importer. The dependence on iron to sustain proliferation and perturbed iron management systems were shown to largely sensitize these stem cells to ferroptotic inducers. Erastin, used to this end, was confirmed to reduce both ovarian tumor number and mass [137]. Ironomycin, a synthetic derivative of salinomycin, was capable of targeting breast cancer stem cells, the maintenance of which was iron-dependent [138]. Pancreatic cancer cells, typically linked to poor clinical prognosis, also proved sensitive to ferroptosis-inducing prodrugs synthesized as hybrids of dihydroartemisinin and derivatives of salinomycin [139]. Both approaches exploited so-called ‘active ferroptosis’ as an inherent vulnerability of cancer stem cells. As opposed to attempting to contain the level of ROS that occur upon iron exposure, they had compounds accumulate in the lysosomes of persister cells and release the lysosomal pool of iron from within. The concept of modulating ferroptosis in cancer stem cells is an understudied niche and an interesting direction to engage bioinorganic compounds with known pro-ferroptotic properties.

## 6. Poisons and Toxicants

One can argue that any chemical can be considered poisonous to humans depending purely on the dose administered. However, certain elements are either obvious xenobiotics in this regard or they occur in the human environment at an absolute minimum. Their therapeutic introduction as inducers of regulated cell death could be seen as controversial and potentially hazardous, because compounds like arsenic or mercury are by themselves a driving force behind various disorders. However, the same disorders caused by adventitious exposure to toxic elements could be treated more effectively with more advanced knowledge of their underlying molecular mechanism. This is where the subject of elements detrimental to the human body becomes relevant with respect to ferroptosis.

In the previous section, the role of zinc was discussed as an inducer of ferroptosis. Zinc occurs as a cofactor in numerous human enzymes of all six main classes and plays either a structural or regulatory role in up to 10% of human proteins [140]. The pro-ferroptotic effects reported upon zinc induction are strikingly similar to what has been reported in the case of arsenite. The trivalent inorganic form of arsenic is a highly toxic 1 group carcinogen [141]. Six-month-long exposure to arsenite in the concentration range of 0.5–5 mg/L drinking water mimicked the level of contamination in the natural environment and caused neuronal cell death and synaptic alterations throughout the hippocampus in mice. The ferroptotic nature of this pathology was confirmed by cell morphological observations, elevated levels of intracellular iron and malondialdehyde and downregulation of SLC7A11 and GPX4. Neuronal-like in vitro cell cultures displayed similar trends upon 24-h induction with 1.5–2.5 μM arsenite. Arsenite-induced ferroptosis was NCOA4-mediated and accompanied by reduced ferritin expression [142], most likely resultant from ferritinophagy rather than inhibited FTH1 translation as was evidenced in the case of zinc oxide induction [124]. The disturbed expression of genes related to Fe homeostasis, including transferrin receptor protein 1 (TfR1), divalent metal transporter 1 (DMT1) and ferroportin (FPN) confirms the key role of iron metabolism despite pro-ferroptotic induction with non-iron compounds [142].

The same type of outcome was observed in the context of arsenite-induced male reproductive toxicity, where testicular cell death was shown to depend on the mechanism of ferroptosis. Several male reproductive diseases are known to depend on the ferroptosis mechanism due to the deposition of iron or ROS in testis as well as the dependency of spermatogenesis on iron abundance [143]. In this case however, 6-month exposure to drinking water contaminated with arsenite as low as 5 mg/L was shown to underlie pathological changes in mouse testis (2.5 μM in the case of GC-2spd cells cultured in vitro). While the body weight of the mice was unaffected, the testicular weight largely decreased. Although no sperm deformation was observed in mice, its number was decreased and its morphology underwent clear alterations, most of which were related to mitochondria [144].

This study is one of many consolidating the general consensus that mitochondria are organelles unambiguously implicated in the progression of ferroptosis. Cellular respiration performed therein is the source of the majority of cellular reactive oxygen species [145]. The emerging significance of endoplasmic reticulum (ER) in ferroptotic signaling is much less explored, but at the same time seems far from a *cul*-*de*-*sac* [146]. The already referred-to ZIP7 mediates zinc translocation from ER [132]. Arsenic, on the other hand, was shown to affect mitochondria-associated ER membranes (MAMs), i.e. junction sites between both organelles [147], which are incidentally indispensable to autophagy process [148]. MAMs dysfunction upon acute exposure to As was reflected by the downregulation of Mfn-2, a mediator of the coupling between mitochondria and ER, as well as elevated PERK phosphorylation, the process of which transduces ER stress signal caused by misfolded proteins overloading the endoplasmic reticulum. Also, both these alterations, attenuated by PERK inhibitor GSK2656157 as well as by overexpression of Mfn-2 plasmid, were shown to repress As-caused ferritin upregulation and to abolish As-induced GPX4 downregulation, leading to the inhibition of As-induced ferroptosis in pulmonary epithelial cells. These results point to MAMs as a potential mediator of the ferroptotic cell death that underlies pulmonary function decline caused by exposure to arsenic [147,148,149].

Arsenic trioxide has attracted a lot of attention in recent months due to its ferroptosis-inducing properties in various cancer cells. It was shown to suppress proliferation of neuroblastoma cells by inhibiting the GSH-GPX4 pathway and activating numerous proteins known to play a role in ferritinophagy and iron overload [150,151]. It also caused rat cardiomyocyte H9c2 cell death, which involved the promotion of oxidative stress and was preventable by ferroptosis inhibitor Fer-1. Although the compound could not be safely applied in acute promyelocytic leukemia treatment due to its toxicity, ferroptosis inhibitors could serve as a potential treatment of cardiotoxicity caused by exposure to arsenic trioxide [152]. A combined therapy against platinum-sensitive ovarian cancer was proposed, comprising arsenic trioxide and PARP (Poly (ADP-ribose) polymerase) inhibitors. The reported outcome revealed suppressed proliferation of human ovarian carcinoma cell lines and of tumor growth in mice xenograft models. Ferroptosis contributed to the effect, as evidenced by i.a. decreased expression of SLC7A11 and GPX4 as well as of SCD1 (stearoyl-CoA desaturase 1) and ACSL3 (acyl-CoA synthetase long-chain family member 3), both governing lipid metabolism, the disturbance of which entails phospholipid peroxidation [153,154].

Mercury chloride was shown to provoke ferroptosis in chicken embryo kidney cells [155] as well as in fish neuronal cells [156]. Nephrotoxic mercury-induced ferroptosis was reported as a dominant type of cell death within the 12-h period following exposure. Mercury-induced NCOA4-mediated ferritinophagy, as was the case with arsenite, along with the upregulation of other ferritinophagy-related proteins: ATG5, ATG7, and LC3B. The degradation of ferritin (FTH more susceptible than FTL subunits) caused intracellular release of iron and an accumulation of ROS formed in the Fenton reaction [155]. Notably, similar results were reported using methylmercury to induce rat primary astrocytes and liver cells, but the loss of FTH1 and iron overload were attributed to the inhibited expression of the storage protein rather than degradation [157]. In a related study, the pro-ferroptotic effect of neurotoxic mercury was evidenced in fish, but the results obtained in vivo could not be reproduced using cultured cells. Rather than directly interfering with iron metabolism, the compound was shown to stimulate gut dysbiosis. Ferroptosis was in this case postulated to underlie brain injuries caused by *Aeromonas hydrophila*, which thrived in the intestinal microenvironment thrown off balance by mercury, indicating an indirect relationship between the metal inducer and cellular death [156].

The inhibition of ferroptosis may also prove therapeutic in the course of neurodegenerative diseases associated with prolonged exposure to lead. Studies conducted both in vitro and in vivo showed that lead acetate induced numerous well-established indicators of ferroptotic cell death, such as iron overload, ROS accumulation, decreased GSH and lipid peroxidation [158]. Gradually, uncharted components of the regulatory mechanism involved in the process of iron-dependent cell death are unveiled in the hope of becoming a target for a novel form of treatment. Among these, multiple non-coding RNAs have been shown to affect the modulation of various genes implicated in ferroptosis. In head and neck cancers alone 926 ferroptosis-related lncRNAs (long non-coding RNAs) were identified serving as scaffolds, molecular signals, guides, or decoys. Some of them are ascribed relevance for prognostic clinical prediction [159]. Another type of molecules includes miRNA, the role of which is to bind mRNA and prevent translation [160]. It was reported that miR-378a-3p miRNA modulated Pb-induced ferroptosis in neuronal cells by suppressing SLC7A11 expression. Inhibition of the nucleic acid molecule, on the other hand, recovered SLC7A11 mRNA expression by 23.88% and partly restored ROS and GSH levels [158]. Notably, the ferroptosis-promoting effect of miR-378a-3p is analogous to that exerted by IFNγ in adrenocortical carcinoma cells. In the latter case, however, the ferroptotic induction was a desired outcome thought to improve the antitumor response in cells resistant to immunotherapy [161]. The aforementioned findings relating to toxic compounds and their effects are recapitulated in Table 4 below.

## 7. Conclusions

The course of ferroptosis is susceptible to multiple bioinorganic modulators, which either exploit cellular vulnerability to ROS and lead towards resultant cell death, or which patch it and render the cell less sensitive to oxidative stress. While some of the discussed compounds have been known before to harness anti-cancer properties, in many cases they have not yet been identified as ferroptotic modulators per se due to the novelty of the concept of ferroptosis as a distinct cell death modality. In recent years, nanotechnology-based techniques of synthesis rediscovered many of the already-established inorganic compounds and played a major role in differentiating their impact as bioactive agents. As a result, the phenomenon of regulated cell death quickly became the focus of diverse attempts aimed at derailing or restoring cellular iron metabolism through the use of simple chemical cues.

As demonstrated in this review, this fledgling area of science holds a lot of promise for future biomedical inventions. Bioinorganic compounds composed of gallium, sulphur, selenium, tellurium, manganese, copper, cobalt, zinc and possibly many other elements, could prove significant as co-therapeutics that are capable of evoking or overturning selective cell death signaling. On the other hand, diseases caused by exposure to toxic arsenic, lead or mercury, if reliant on ferroptotic mechanism, could be successfully tackled with the use of molecules inhibiting iron-dependent cell death. As of today, the list of ferroptosis-associated anti-cancer drugs tested in preclinical experiments or clinical trials is short and notably includes the H_2_O_2_/Fe_3_O_4_-PLGA nanosystem, where bioinorganic iron(II,III) oxide nanoparticles are used as a reagent to trigger a Fenton reaction [162]. Other than that, the potential use of ferroptosis-based therapies, including the use of bioinorganic modulators, remains at an experimental stage of research despite the flourishing scientific progress made to date. Fourteen years after the first published report on ferroptosis, it becomes an emerging challenge to specifically target cancer stem cells, known for their lethality and refractory profile in response to traditional drugs. As a means of addressing this, ferroptosis seems to have every chance of closing a loophole in the relentless attempts to bypass the problem of therapeutic resistance.

## Figures and Tables

**Table 1:** Recapitulation of the gallium-based compounds and their modulatory effect on ferroptosis-related cell survival and metabolism.

Bioinorganic Modulator	Research Model	Outcome	Ref.
Gallium nanoparticles +low level of gamma radiation	Diethylnitrosamine (DEN)—induced hepatocellular carcinoma (HCC) in rats Human hepatocellular carcinoma (HepG2) cell line	Reduced serum levels of AST, ALT, AFP, GGT, MM9, TGF-β in HCC Cytotoxic effect in HepG-2, IC_50_ 8.0 μg/mL	[66]
Gallium nanoparticles coated by gallic acid	Diethylnitrosamine (DEN)—induced hepatocellular carcinoma (HCC) in rats Human hepatocellular cancer (HepG-2) cell line	Cytotoxic effects at the interface of primary hepatocarcinogenesis, key hallmarks of cancer diminished (normalized liver C-Myc, HSP-70, and Cyt-c mRNA expression, ameliorated iron accumulation), reduced liver function indices ALP, ALT, AST Strong cytotoxic effect in HepG-2, IC_50_ 0.71 ± 0.02 µg/mL	[68]
Gallium nanoparticles coated by ellagic acid	7, 12 dimethyl benz[a] anthracene (DMBA)—induced mammary gland carcinogenesis in rats Human breast cancer (MCF-7) cell line	Reduced serum levels of ALT, AST, urea, and creatinine, decreased MDA, increased SOD, GSH, total iron binding capacity, mild recurrence of normal histopathological appearance Cytotoxic effect in MCF-7, IC_50_ 2.86 ± 0.3 μg/mL	[75]
Gallic acid-gallium polyvinyl pyrrolidone nanoparticles	CP—induced and ischemia-reperfusion injury—induced acute kidney injury in mice Cisplatin (CP)—induced ferroptosis in HK-2 cells	Protection against renal ischemia-reperfusion injury in mice Reduced accumulation of intracellular free iron and mitochondrial dysfunction, suppressed parameters of ferroptosis (attenuated iron accumulation, inhibited ferritinophagy, restored GPx4 and NCOA4 expression)	[79]
Gallium(III) complex with cloxyquin	Human rhabdomyosarcoma cells	Killed bulk and stem rhabdomyosarcoma cells, IC_50_ 1.3 ± 0.1 and 6–8 ± 2 μM, respectively Induced ferroptosis, significant increase in ROS level, reduced GPx4, stimulated expression of TfR1, potentiated accumulation of lipid peroxides Coincubation with ferrostatin reduced the potency of the compound	[80]

**Table 2:** Recapitulation of the chalcogen-based compounds and their modulatory effect on ferroptosis-related cell survival and metabolism.

Bioinorganic Modulator	Research Model	Outcome	Ref.
In situ generated hydropersulfides from cumyl-SSH	(1S,3R)-RSL3—induced ferroptosis in Pfa1 mouse embryonic fibroblasts	Restored cell viability in a dose-dependent manner, IC_50_ 6.2 μM Ferroptosis previously induced through inactivation of GPx4 or deletion of the gene encoding it was suppressed	[83]
mPEG-PLG(DNs), i.e., amphiphilic polymer prodrug of sulfur dioxide	Human gastric cancer cell line MKN-1 Human gastric mucosa cell line GES-1	GSH depletion and SO_2_ generation resulting in amplified oxidative stress accelerated and lipid peroxidation in malignant cells, downregulated the expression of GPx4 in malignant cells IC_50_ 240.9 ± 26.3 μg/mL in MKN-1 IC_50_ 440.9 ± 44.7 μg/mL in GES-1	[86]
Sodium selenite	Human cancer cell lines: MCF-7, PC3, U87MG, HT-1080, HeLa, HCT-116 and A172 cells Cytotoxicity testing: normal human fetal glial cells (SVG P12) vs. U87MG human malignant glioma cells	Reduced cell viability Downregulated SLC7A11, GSH, GPx4 Increased iron accumulation and lipid peroxidation Preferentially cytotoxic to malignant cells	[91]
Selenate and selenite transformed into selenomethionine	Zebrafish embryos	Disrupted mitochondrial morphology, elevated ROS-induced oxidative stress, significant increase in MDA, decrease in GSH, ferroptosis in embryonic eye cells At 24 h post fertilisation: gpx4a and gpx4b up-regulated, while slc7a11 down-regulated At 96 h post fertilisation: gpx4a and gpx4b decreased, slc7a11 unchanged relative to the controls	[95]
Sodium selenite, and sodium selenate	N27 immortalized neuronal cell line	anti-ferroptotic, protective against erastin- and RSL3-induced ferroptosis, increase in GPx1 and GPx4 expression Selenite was the most potent inhibitor of ferroptosis with EC_50erastin_: 38 nM	[97]
Sodium selenite	Rat model of T10 vertebral contusion injury	Decreased iron concentration and levels of the lipid peroxidation, increased the protein and mRNA expression of Sp1 and GPx4, promoted survival of neurons and oligodendrocytes, inhibited proliferation of astrocytes	[99]
Sodium selenite	8–10 week old Male C57BL/6 mice HT22 murine hippocampal cells cultures Human HT-1080 fibrosarcoma cells	Dose-dependent neuroprotective efect against ferroptotic death in transformed and non-transformed cells, induction of selenoproteins (incl. GPx4), activation of the transcription factors i.a. TFAP2c and Sp1	[98]
Cadmium telluride quantum dots	RAW264.7 macrophages	Ferroptotic death as a result of imbalanced oxidative and antioxidant systems, associated with decrease in NRF2, phosphorylation of ERK1/2, activated ferritinophagy	[105]

**Table 3:** Recapitulation of the transition metal-based compounds and their modulatory effect on ferroptosis-related cell survival and metabolism.

Bioinorganic Modulator	Research Model	Outcome	Ref.
Manganese carbonate nanocubes	HeLa cells Tumor bearing mice	Upregulated intracellular ROS was prompted by Mn^2+^ ions and contributed to antitumor effect	[111]
Manganese dioxide-coated mesoporous silica nanoparticles	U87MG human glioma cells	Decline of antioxidant defense resulting from GSH depletion, ROS generation prompted by Mn^2+^ ions, dose- and time-dependent toxicity of Mn^2+^ ions	[112]
Arginine-rich manganese silicate nanobubbles	Huh7 liver cancer cells Liver L02 normal cells Huh7 tumor xenograft nude mice	Upregulation of reactive oxygen species, GSH depletion, inactivation of GPx4	[113]
Cisplatin prodrug-loaded manganese-deposited iron oxide nanoplatform	HeLa cells Tumor-bearing BALB/c-Nude mice	Ferroptotic effect through generation of reactive oxygen species, reduced cell viability, high levels of intracellular lipid peroxide, significantly lowered GSH, prominent anti-tumor efficacy in vivo	[115]
Copper–amino acid mercaptide nanoparticles	Human cervical carcinoma (HeLa) cells, human prostate cancer (PC-3) cells, human adipose-derived stem cells (hADSCs), human renal tubular epithelial (HK-2) cells, human bone mesenchymal stem cells (hbMSCs), human breast cancer cells (MCF-7), doxorubicin-resistant MCF-7 cells (MCF-7R)	Anti-cancer effect based on GSH depletion, generation of reactive oxygen species, low systematic toxicity, selective cytotoxicity, tumor suppression at ∼72.3% inhibition rate	[117]
Copper/Manganese Silicate Nanospheres	MCF-7, A549 and NHDF cells MCF-7 cancer-bearing female Balb/c mice	Upregulation of reactive oxygen species, GSH depletion, disrupted tumor microenvironment through relief of intracellular hypoxia, cancer cells growth significantly inhibited	[118]
Cu^2+^ (atsm)	Primary cortical neurons Immortalised neuronal cell lines, N27	EC_50_ ≈ 130 nM Cytoprotective effect against lipid peroxidation and ferroptotic lethality	[119]
Copper sulfate	AsPC-1, PANC-1, MIA PaCa-2, and SW 1990 cell lines Mouse xenograft model	Amplified cell death and lipid peroxidation, GPx4 autophagy	[120]
Zinc oxide nanoparticles	HUVECs and EA.hy926 cells	Macroautophagy/autophagy-dependent NCOA4-mediated ferroptosis	[124]

**Table 4:** Recapitulation of the toxic compounds and their modulatory effect on ferroptosis-related cell survival and metabolism.

Bioinorganic Modulator	Research Model	Outcome	Ref.
Arsenite	Neuronal-like PC-12 Adh cells C57BL/6J mice	Decreased levels of ferritin and NCOA4, increased autophagy marker LC3B and lipid peroxidation, elevated iron content, SLC7A11 and GPX4 expression declined Neuronal ferroptotic cell death in the hippocampus via activation of ferritinophagy, pathological changes in the mitochondria	[142]
Arsenite	C57BL/6J mice GC-2spd cells	Pathological changes in mouse testis, reduced number of sperm, mitochondrial oxidative damage Reduced cell viability, accumulation of iron, production of reactive oxygen species, lipid peroxidation, reduced GPX4 and SCL7A11 expression	[144]
Arsenic trioxide	SK-N-BE(2) neuroblastoma cell line	Inhibited proliferation, reduced viability, ROS and iron accumulation, ferritinophagy, downregulated GPx4	[151]
Arsenic trioxide	Rat cardiomyocyte H9c2 cells	Reduced viability, IC_50_ 20.7 μM production of ROS, loss of the mitochondrial membrane potential, distortion and enlargement of myocardial mitochondria, hyperactive ER stress, impaired autophagy, accumulation of MDA, depletion of GPx-4	[152]
Arsenic trioxide +olaparib	Platinum-resistant A2780-CIS and SKOV3-CIS cell lines BALB/c mice	Suppressed cell proliferation and loss of viability indicating synergetic effects, increased lipid peroxidation, significant reduction of formed colonies, increased MDA, decreased SLC7A11and GPx4 levels, ferroptotic cell death and suppressed tumor growth	[153]
Mercuric chloride	Chicken embryo kidney cells	Ferroptosis followed by necroptosis, ferritinophagy, ROS generation, iron overload	[155]
Mercuric chloride	Common carp	Mitochondrial defects, increased levels of iron and MDA, depleted GSH, Brain injury and memory loss, gut microbiota disorders	[156]
Lead acetate	C57 mice HT22 hippocampal neuronal cell line	Decreased cell viability related to Pb-promoted iron overload, accumulation of ROS and MDA, depleted GSH, downregulated expression of SLC7A11 Ferroptosis reported in brain tissues (hippocampus, cortex, hypothalamus, striatum)	[158]

## Data Availability

Not applicable.
